# Impact of Thermal Processing and Storage on the Quality and Chirality of Linalool in Blueberry Juice

**DOI:** 10.3390/molecules31183198

**Published:** 2026-09-10

**Authors:** Zeyu Zhou, Chaoyi Tu, Fang Yuan

**Affiliations:** 1 College of Food Science and Technology, Huazhong Agricultural University, Wuhan 430070, Chinatucy1212@163.com (C.T.); 2 Hubei Key Laboratory of Fruit & Vegetable Processing & Quality Control, Huazhong Agricultural University, Wuhan 430070, China

**Keywords:** blueberry juice, thermal treatment, storage conditions, chiral volatile compounds, linalool enantiomers

## Abstract

Blueberry juice is valued for its distinctive flavor and health-promoting bioactive compounds, but thermal processing and subsequent storage can alter its quality and aroma. While previous studies have focused on total volatile profiles, the behavior of chiral aroma compounds—particularly linalool enantiomers—during processing and storage remains poorly understood. This study aimed to investigate the effects of pasteurization (PT, 90 °C for 30 s) and ultra-high temperature processing (UHT, 135 °C for 6 s) on the physicochemical properties, bioactive compounds, antioxidant activity, volatile profiles, and sensory characteristics of blueberry juice during storage at 4, 25, and 35 °C for 4 weeks, with a special emphasis on the enantiomeric changes in linalool. A chiral column-based GC-MS method was established to quantify (R)- and (S)-linalool enantiomers, given their distinct odor thresholds and sensory contributions. The results showed that both thermal treatments ensured microbial safety, but UHT caused greater color deterioration, loss of phenolics and anthocyanins, and formation of off-flavor compounds. PT better preserved color, bioactive components, and natural fruity aroma. Storage temperature was the dominant factor driving quality decline, with 4 °C significantly retarding deterioration. Notably, the two thermal processes exhibited distinct mechanisms affecting chiral linalool stability: PT primarily induced isomerization of (R)-linalool to the (S)-form, leading to a gradual decrease in the R/S ratio, whereas UHT led to direct degradation of (R)-linalool, resulting in a more rapid shift in the R/S ratio under the same conditions. These findings highlight the importance of monitoring enantiomeric composition rather than total linalool content for flavor quality assessment. The combination of PT and refrigerated storage (4 °C) is recommended to maximize overall quality retention, as PT better preserves the natural chiral balance of linalool. This study demonstrates that appropriate thermal processing and low-temperature storage are effective strategies to maintain the quality and chiral flavor stability of blueberry juice, providing new insights into the role of enantiomer-specific changes in processed fruit products.

## 1. Introduction

Due to their unique flavor and nutritional value, blueberry fruits and products are highly appreciated by consumers worldwide, and the global production has been expanding annually [1]. Blueberries are recognized as a rich source of anthocyanins, phenolics, and antioxidants, which contribute to various health benefits [2]. However, fresh blueberries are highly perishable and have a limited harvesting season. To extend availability and reduce postharvest losses, a large proportion of blueberries is processed into juice, jam, and other products. Among these, blueberry juice is the most common product, valued for its convenience and retained nutritional properties [3].

Thermal processing technologies, particularly pasteurization (PT) and ultra-high temperature processing (UHT), is the most widely used method to ensure microbial safety and extend the shelf life of blueberry juice [4]. Although non-thermal technologies offer theoretical advantages in preserving heat-sensitive components, their industrial application remains limited [5,6,7]. Therefore, optimizing thermal processing parameters and understanding their impact on juice quality remain practically relevant for the blueberry juice industry.

The aroma of blueberry juice is a critical quality attribute that influences consumer acceptance. More than 100 volatile compounds have been identified in blueberries, including esters, aldehydes, terpenes, and alcohols [8]. Among these, terpenes such as linalool and α-terpineol are considered important contributors to the characteristic blueberry aroma [9,10]. However, compared with strawberries and raspberries, which have over 360 and 200 reported volatile compounds [11], respectively, research on blueberry aroma is relatively limited. Furthermore, most existing studies focus on total volatile profiles, while the behavior of chiral volatile compounds—particularly linalool enantiomers—during thermal processing and storage has received little attention. Enantiomers of the same compound can exhibit markedly different odor thresholds and sensory contributions. For instance, specific enantiomeric distributions of linalool have been reported in various food systems, including coffee, beer, honey, and iced tea, with (R)-linalool exhibiting woody, green, and cooling aroma notes (odor threshold: 0.8 μg/L) and (S)-linalool characterized by sweet and floral notes (odor threshold: 7.4 μg/L) [12,13]. The substantial differences in both odor thresholds and sensory profiles between the two enantiomers result in their distinctly different contributions to the overall flavor of foods. In blueberries, (R)-(−)-linalool has a much lower odor threshold than its (S)-(+)-counterpart [14]. Changes in the enantiomeric ratio during processing and storage could therefore significantly affect the overall flavor quality.

Headspace solid-phase microextraction combined with gas chromatography-mass spectrometry (HS-SPME-GC-MS) is a widely applied technique for volatile analysis in blueberry studies [15]. For chiral separation, cyclodextrin-based capillary columns such as Cyclosil-B have been successfully applied to resolve linalool enantiomers in various food matrices [13]. However, the application of chiral GC-MS to blueberry juice under different thermal and storage conditions has not been systematically explored.

Post-processing storage is another critical factor influencing juice quality [16]. Storage temperature and duration govern the stability of bioactive compounds, color, and volatile profiles. Studies on orange juice, apple juice, and pomegranate juice have demonstrated that elevated storage temperatures accelerate the degradation of key aroma compounds and promote the formation of off-flavors via Maillard, Strecker, and acid-catalyzed reactions [17,18,19]. For blueberry juice, how thermal history (PT vs. UHT) and subsequent storage temperature jointly modulate quality indices and chiral volatile stability remains largely unknown. Based on our literature search, no study has systematically investigated the combined effects of thermal processing and storage conditions on the enantiomeric behavior of linalool in blueberry juice.

Basic physicochemical parameters such as pH and total soluble solids (TSS) are fundamental indicators of juice quality, reflecting the stability of the acid–base balance and soluble constituents [20,21]. Total phenolic content (TPC) and total anthocyanin content (TAC) are routinely measured as key bioactive markers due to their established roles in antioxidant capacity and color attributes [22,23]. The DPPH radical scavenging assay was employed as a preliminary screening method for antioxidant activity, offering a simple and reproducible assessment of the overall reducing potential [24]. Although more specific analyses (such as HPLC-based quantification of individual phenolic compounds and anthocyanins) could provide finer resolution, the selected set of parameters allows a comprehensive yet efficient evaluation of the major quality dimensions affected by thermal processing and storage [25].

Therefore, in this study, we investigated the quality changes in blueberry juice subjected to PT and UHT treatments during storage at 4, 25, and 35 °C for 4 weeks. Clarified juice was used to eliminate the interference of suspended particles on chiral GC–MS analysis and to ensure uniform heat transfer during thermal treatments. A chiral GC-MS method based on a Cyclosil-B column was established to quantify (R)- and (S)-linalool enantiomers. The physicochemical properties, bioactive compounds, antioxidant activity, and volatile composition were monitored. The aim of this study was to provide a comprehensive understanding of the quality dynamics and chiral flavor stability of thermally processed blueberry juice during storage, and to propose an optimal processing–storage strategy for maximizing quality retention.

## 2. Results and Discussion

### 2.1. Effects of Thermal Processing on pH and Total Soluble Solids of Blueberry Juice During Storage

pH and TSS are core physicochemical indicators for evaluating juice quality [17]. As shown in Table 1, pH values remained stable within a narrow range of 3.24–3.36 throughout the 4-week storage period, with no significant differences between PT and UHT treatments at any temperature (*p* > 0.05). This indicates that the acid–base environment of blueberry juice is minimally affected by the type of thermal treatment, and the stability can be attributed to the good buffering capacity of organic acids naturally present in blueberry juice, such as citric acid and malic acid [26]. Storage temperature, however, exerted a significant effect on pH (*p* < 0.001): pH at 35 °C was significantly lower than at 4 °C and 25 °C (*p* ≤ 0.003), while no difference was found between 4 °C and 25 °C. This slight pH decrease at elevated temperature may reflect the accelerated formation of organic acids (e.g., formic acid, acetic acid) from sugar degradation pathways [25], although the magnitude of change is small and does not compromise the acidity quality of the juice.

TSS remained highly stable at 4 °C (fluctuating around 10.5 °Brix, retention > 99%), while a gradual decline was observed at 25 °C (to approximately 10.2 °Brix) and a more pronounced decrease at 35 °C (to approximately 9.75 °Brix, retention still > 93%). Storage temperature significantly affected TSS (*p* < 0.001), with all pairwise comparisons being significant (*p* < 0.001). The TSS decline at higher temperatures likely reflects sugar consumption in non-enzymatic browning reactions, such as caramelization and Maillard reactions [27], which is consistent with the subsequent observations of color changes and furfural accumulation. Importantly, no significant differences in either pH or TSS were detected between PT and UHT treatments at any temperature or time point (*p* > 0.05), indicating that the basic physicochemical matrix of blueberry juice is highly resilient to the thermal loads applied, and that the two thermal treatments did not cause distinguishable changes in pH or TSS. From this, it can be inferred that the quality differences between PT and UHT during storage—such as color, aroma, and bioactive compound retention—arise primarily from the differential responses of more sensitive components, including anthocyanins and volatile compounds [17], rather than from alterations in the fundamental physicochemical properties.

### 2.2. Effects of Thermal Processing Treatments on Color of Blueberry Juice During Storage

**Figure 1:**
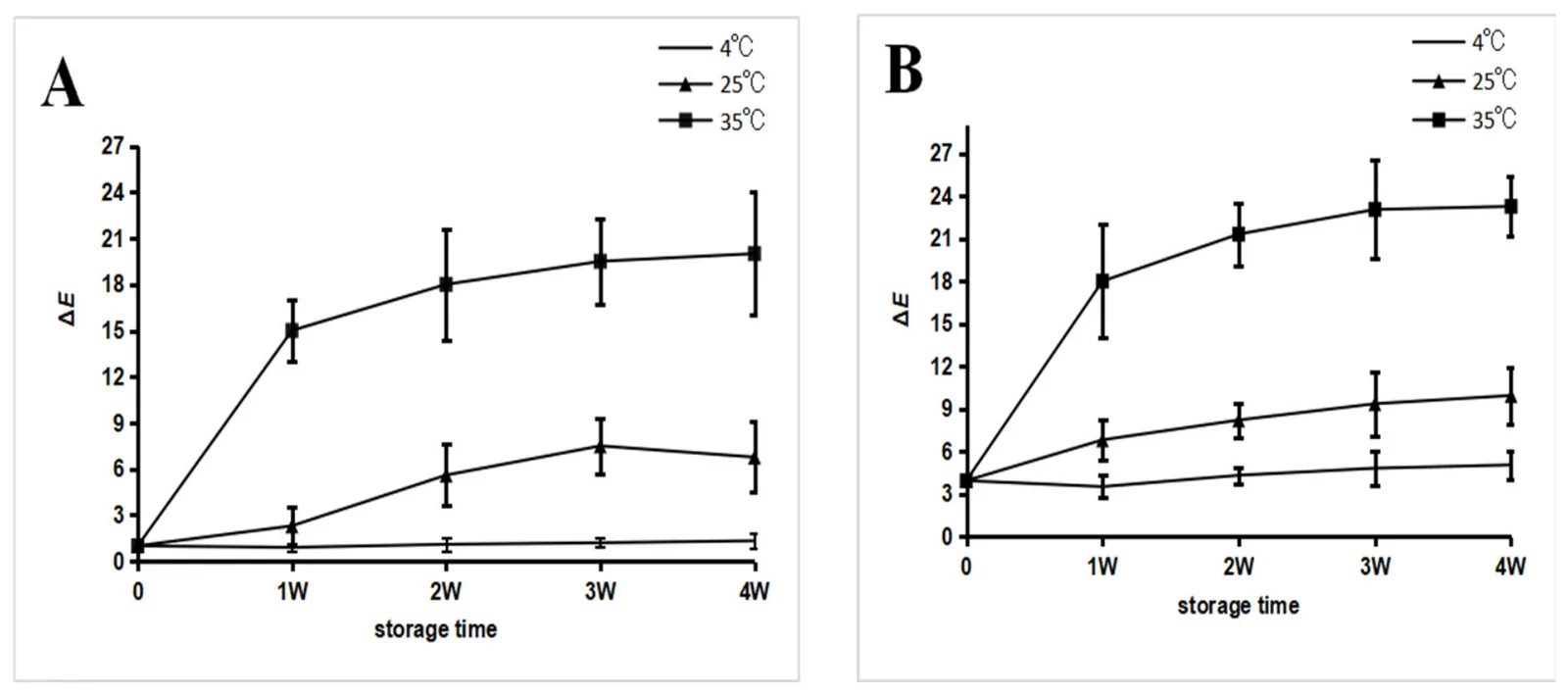
Effects of UHT (**A**) and PT (**B**) on the color (ΔE) of blueberry juice during storage.

Color is an important sensory quality attribute, and total color difference (ΔE) reflects overall color changes during storage [28]. As shown in Figure 1, ΔE values increased over time under all conditions. At 4 °C after 4 weeks, ΔE for PT-treated juice was 1.33 ± 0.33—below the perceptibility threshold of 3—while UHT-treated juice reached 5.03 ± 1.02. At 25 °C, ΔE values were 6.77 ± 1.54 (PT) and 8.42 ± 2.03 (UHT). At 35 °C, ΔE increased sharply during the first week before leveling off, indicating that color-degrading reactions occur rapidly upon exposure to high temperature.

The greater color stability of PT juice can be attributed to its milder thermal load (90 °C, 30 s), which limits anthocyanin polymerization and browning pigment formation compared to UHT (135 °C, 6 s) [29,30]. The sharp ΔE rise at 35 °C aligns with the rapid anthocyanin degradation observed in Section 2.3, confirming that high temperature accelerates both pigment breakdown and non-enzymatic browning. Anthocyanin degradation was linked to color deterioration (ΔE), as evidenced by the parallel trends across all storage conditions. Additionally, the consistently low pH (3.24–3.36) likely helped slow anthocyanin degradation, as anthocyanins are most stable under acidic conditions [31]. Overall, PT is more favorable than UHT for maintaining color stability during storage, particularly when combined with refrigeration.

### 2.3. Effects of Thermal Processing Treatments on Bioactive Compounds and Antioxidant Activity of Blueberry Juice During Storage

As presented in Table 2, PT-treated juice retained significantly higher levels of anthocyanins, total phenolics, and total flavonoids than UHT-treated juice under identical storage conditions. At 4 °C after 4 weeks, anthocyanin content in PT juice was 9.42 mg CGE/100 mL versus 7.92 mg CGE/100 mL in UHT juice (*p* < 0.01), corresponding to retention rates of 72.35% and 68.35%, respectively. Total phenolic retention followed a similar pattern (80.20% vs. 77.99%), and total flavonoid content was also significantly higher in PT juice (39.21 vs. 33.18 mg RE/100 mL, *p* < 0.01). At 25 °C, the same trend persisted with lower absolute values. At 35 °C, degradation was severe across both treatments, with anthocyanins, phenolics, and flavonoids declining to 6.08–7.47, 32.26–33.01, and 25.27–29.92 mg/100 mL, respectively, by week 4.

The superior retention of bioactive compounds in PT-treated juice is attributable to its milder thermal treatment, which minimizes phenolic degradation while effectively inactivating polyphenol oxidase, thereby reducing both thermal and enzymatic losses. Similar observations have been reported in strawberry and grape juice studies, where pasteurization resulted in higher phenolic retention than UHT [32,33]. Interestingly, DPPH scavenging activity showed no consistent treatment-dependent advantage between PT and UHT across storage conditions, suggesting that antioxidant capacity is not solely dependent on anthocyanin and phenolic content [30]. Other factors—such as ascorbic acid or synergistic interactions among antioxidants—may also contribute. Future studies incorporating complementary assays such as ABTS and FRAP would provide a more comprehensive characterization.

### 2.4. Effects of Thermal Processing Treatments on Volatile Compounds of Blueberry Juice During Storage

**Figure 2:**
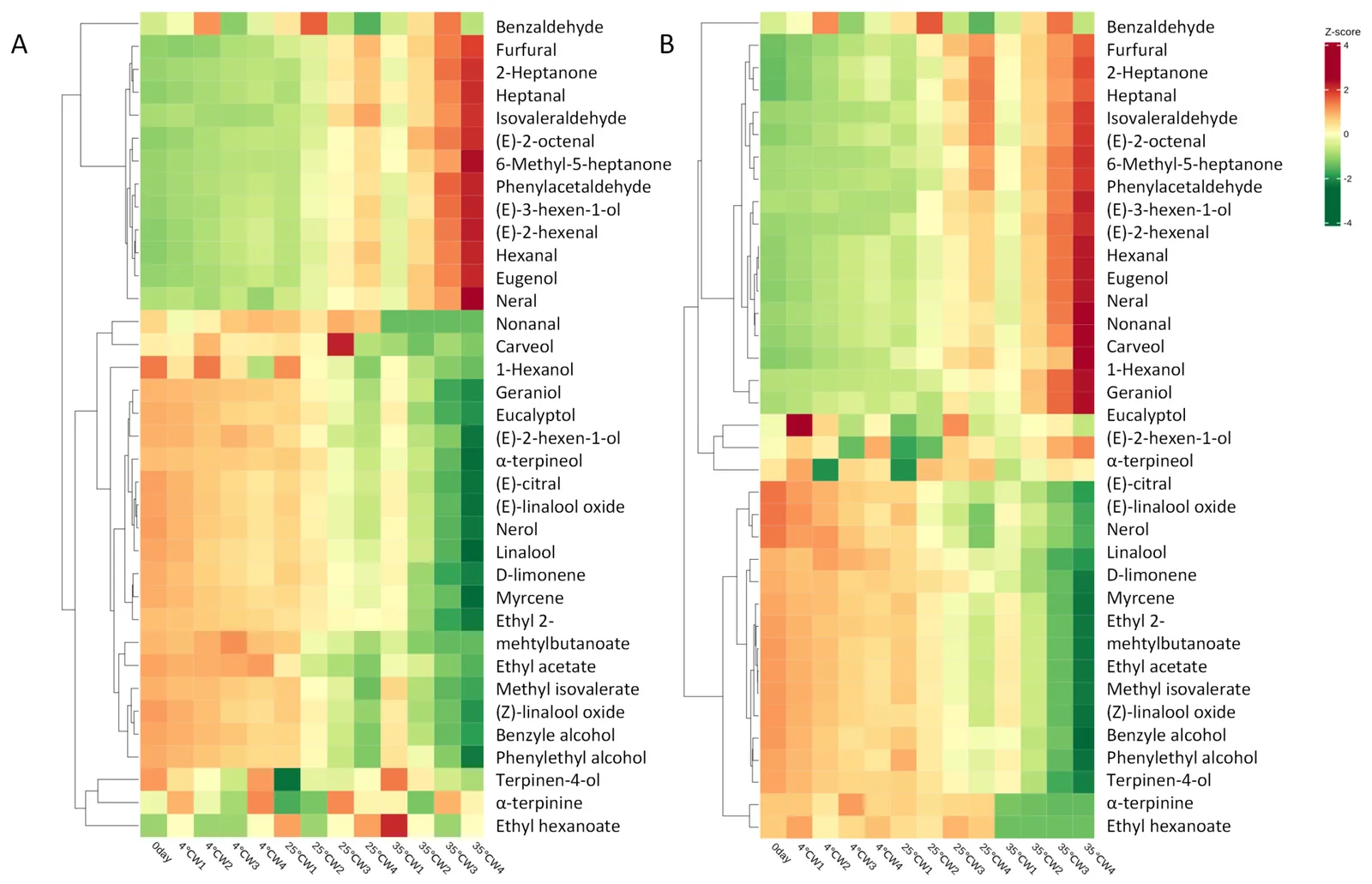
Heatmap and cluster analysis of volatile compounds in blueberry juice after UHT (**A**) and PT (**B**) treatments.

**Figure 3:**
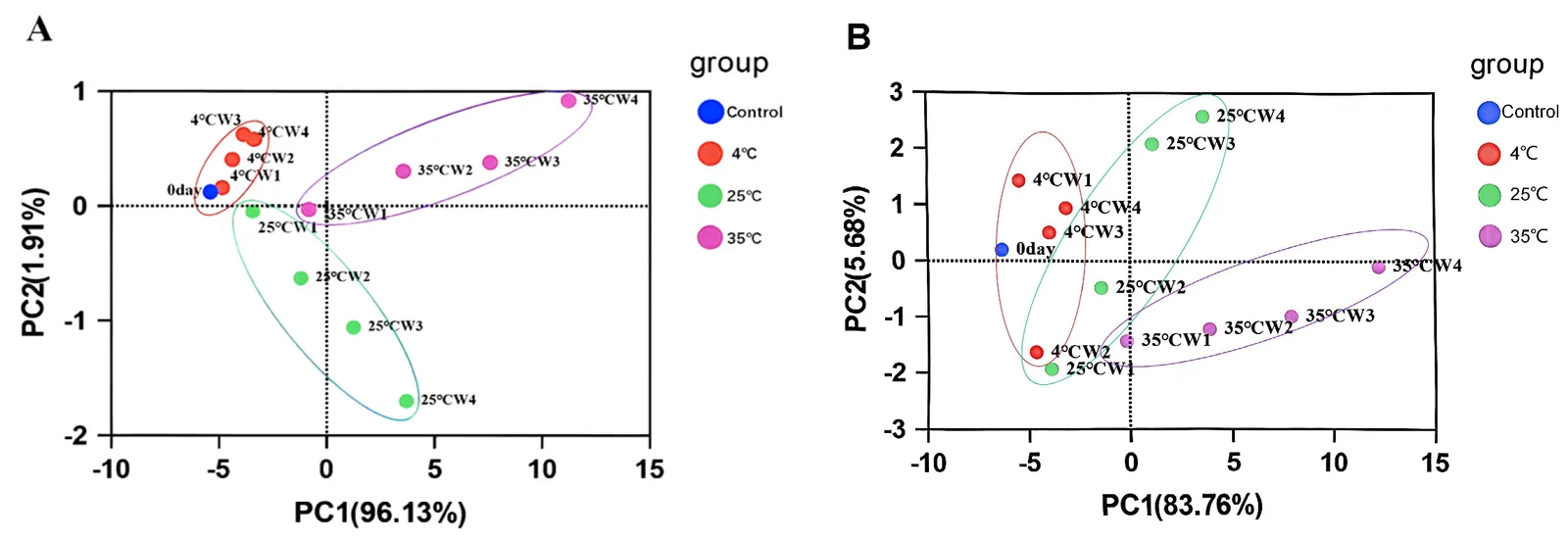
Principal component analysis of volatile compounds in stored blueberry juice after UHT (**A**) and PT (**B**) treatments.

A total of 36 volatile compounds were identified and quantified by HS-SPME-GC-MS. Hierarchical clustering analysis (Figure 2A) revealed two major clusters: Cluster I, comprising compounds such as 1-hexanol, geraniol, and eucalyptol, was more abundant in samples stored at 4 °C and at early storage times; Cluster II, mainly consisting of aldehydes such as nonanal, heptanal, and eugenol, increased significantly after 3–4 weeks at 35 °C. PCA (Figure 3) showed clear separation among storage temperatures, with the first two PCs explaining over 89% of the variance. Samples at 4 °C clustered closest to initial samples, while those at 35 °C deviated most, confirming that low temperature best preserves the original flavor profile. The most notable changes were the accumulation of furfural and the decline in terpenes and esters. Furfural, a typical off-flavor marker derived from sugar dehydration and Maillard reactions, accumulated more in UHT-treated juice than in PT-treated juice throughout storage, and this accumulation was closely related to the lower sensory scores of UHT samples stored at 35 °C. It has been reported in orange juice that furfural accumulation is closely correlated with sensory deterioration [34], which is consistent with our sensory results. The higher accumulation of furfural in UHT-treated juice is due to its more intense thermal load (135 °C for 6 s), which enhances Maillard reactions and sugar dehydration. Gao et al. [35] reported that UHT-treated and pasteurized juices had significantly higher levels of furfural compounds compared to high-pressure processing (HPP)-treated juices. These furanic aldehydes, responsible for cooked and burnt flavor notes, contribute to the lower sensory scores and off-flavor perception observed in UHT juice, especially when stored at 35 °C.

Esters such as ethyl acetate and ethyl hexanoate, key contributors to fruity aroma [15,36], decreased during storage, especially at higher temperatures. C6 aldehydes, such as hexanal and (E)-2-hexenal, are typically generated from fatty acid oxidation via the lipoxygenase pathway [36,37]. For example, 1-hexanol rose from 99.24 ± 2.38 μg/L initially to 180 ± 3.21 μg/L at 35 °C after 4 weeks, while (E)-2-hexenal increased from 76.17 ± 2.11 μg/L to 198.04 ± 3.21 μg/L (Tables S1–S3). Conversely, linalool content decreased markedly during storage, consistent with its known susceptibility to oxidation and acid-catalyzed degradation in acidic juice matrices [13,38]. The decline in fruity esters combined with the accumulation of aldehydes and furfural led to a loss of characteristic blueberry flavor and the emergence of off-odors described as “cooked”, “stale”, and “burnt”. Cold storage (4 °C) effectively suppressed these undesirable changes, underscoring the importance of refrigeration for maintaining fresh flavor.

### 2.5. Effects of Thermal Processing Treatments on Chiral Linalool Enantiomers

**Figure 4:**
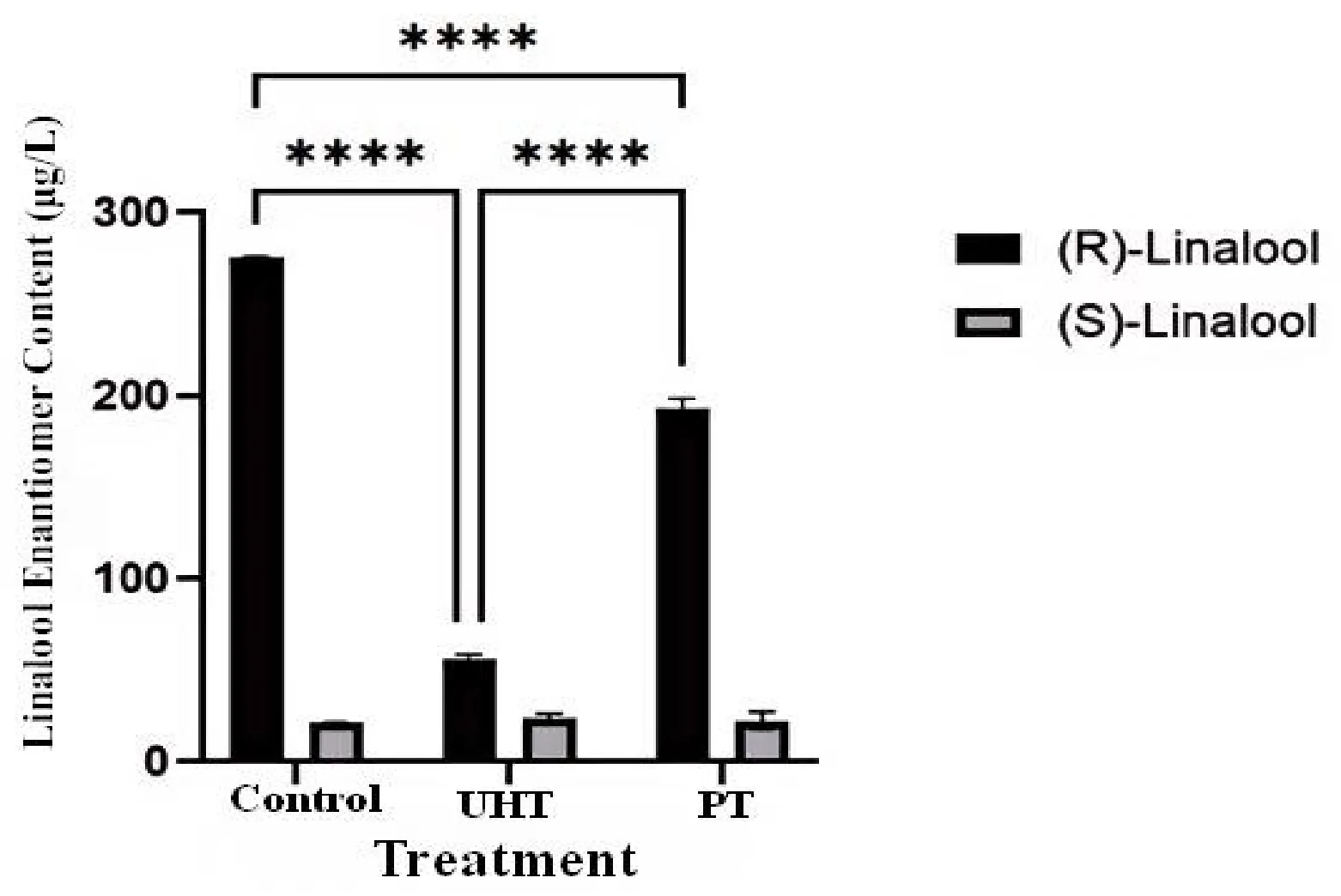
Linalool enantiomer contents after thermal processing treatments. **** indicates a statistically significant difference at *p* < 0.0001.

**Figure 5:**
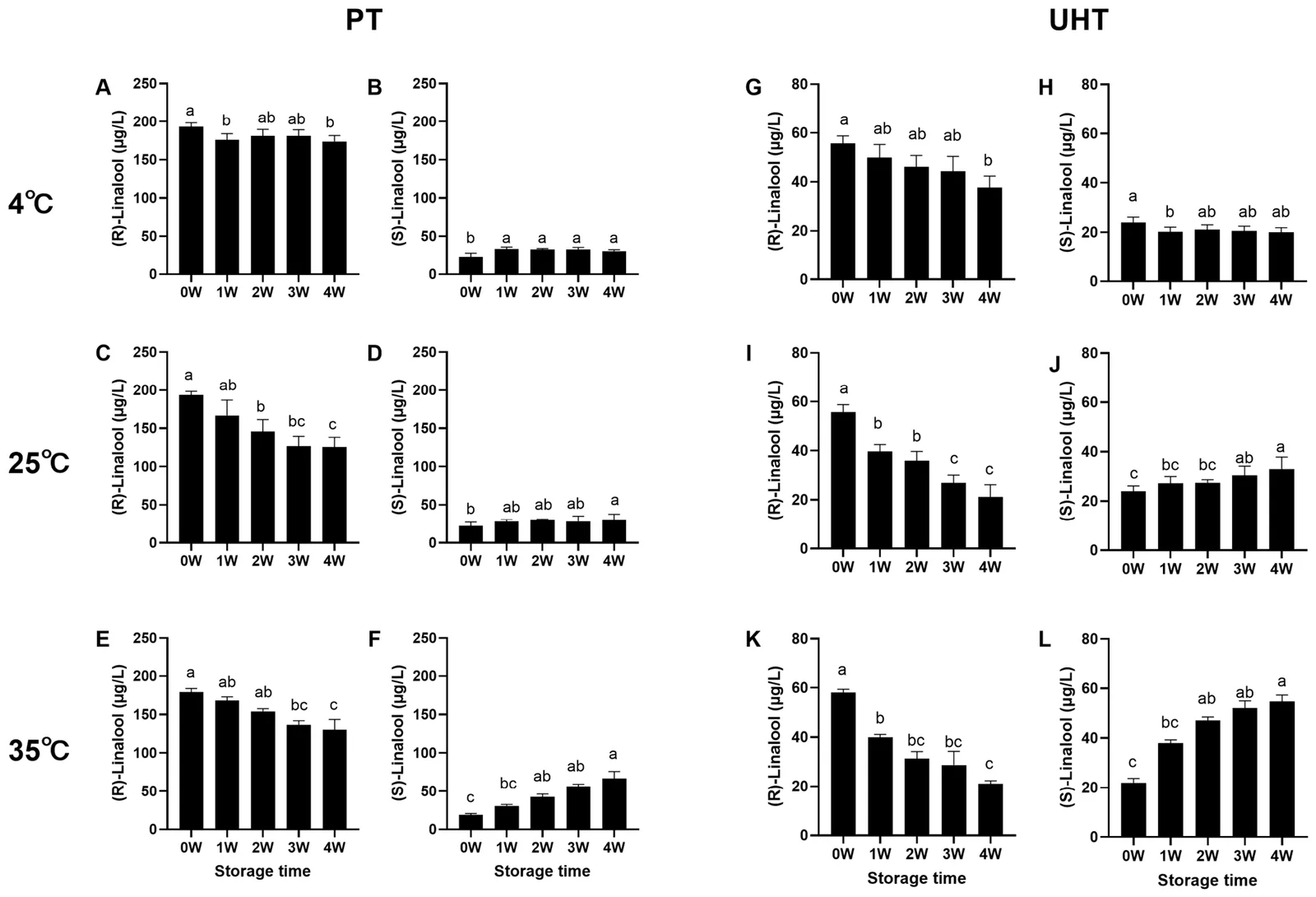
Enantiomeric changes in linalool during storage at 4, 25, and 35 °C after PT (**A**–**F**) and UHT (**G**–**L**). Different letters above the bars indicate significant differences among groups (*p* < 0.05).

(R)-Linalool and (S)-linalool were quantified using an external standard method (Figure 4). Chiral analysis revealed that (R)-linalool was the predominant enantiomer in all samples, and thermal treatment significantly affected its content (*p* < 0.05) but not that of (S)-linalool (*p* > 0.05) (Figure 4). In PT-treated juice, total linalool, that is, the sum of (R)- and (S)-linalool, decreased slightly (from 300.1 to 206.9 μg/L; Figure 4), while the proportion of (S)-linalool increased marginally (from 6% to 10%; Table 3), suggesting partial acid-catalyzed isomerization of (R)-linalool to the (S)-form under mild heating [39]. In UHT-treated juice, total linalool decreased substantially (to 72.4 μg/L; Figure 4) and the R/S ratio dropped sharply (Table 3), indicating that the higher thermal load caused extensive degradation of (R)-linalool, with isomerization playing a secondary role [14]. During storage, the R/S ratio declined progressively with increasing temperature and time (Table 3), most markedly in UHT-treated juice, where it fell to 67:33, 36:64 and 27:73 after 4 weeks at 4, 25 and 35 °C, respectively (Figure 5G–L). During storage (Figure 5), temperature emerged as the dominant factor governing chiral stability [12]. At 4 °C, both enantiomers remained relatively stable. At 25 °C, (R)-linalool decreased while (S)-linalool increased, driving a decline in the R/S ratio through both degradation and isomerization. At 35 °C, the most extreme changes were observed: the R/S ratio in UHT-treated juice shifted from 50:50 at week 0 to 27:73 at week 4, while PT-treated juice showed a less severe shift from 83:17 to 62:38. These results indicate that PT combined with low-temperature storage is more effective at preserving the natural chiral balance of linalool. Similar chiral conversion patterns have been documented in lemon-flavored beverages and juniper spirits [38].

The shift in the (R)/(S) ratio has implications beyond total linalool content. (R)-Linalool has an odor threshold 8–10 times lower than (S)-linalool [27]; therefore, its preferential degradation under UHT and high-temperature storage disproportionately diminishes the perceived intensity of woody and green notes, while the relative increase in (S)-linalool cannot fully compensate due to its higher odor threshold [40]. Monitoring the enantiomeric ratio is thus essential for accurate flavor quality assessment.

### 2.6. Effects of Thermal Processing Treatments on Sensory Quality of Blueberry Juice During Storage

**Figure 6:**
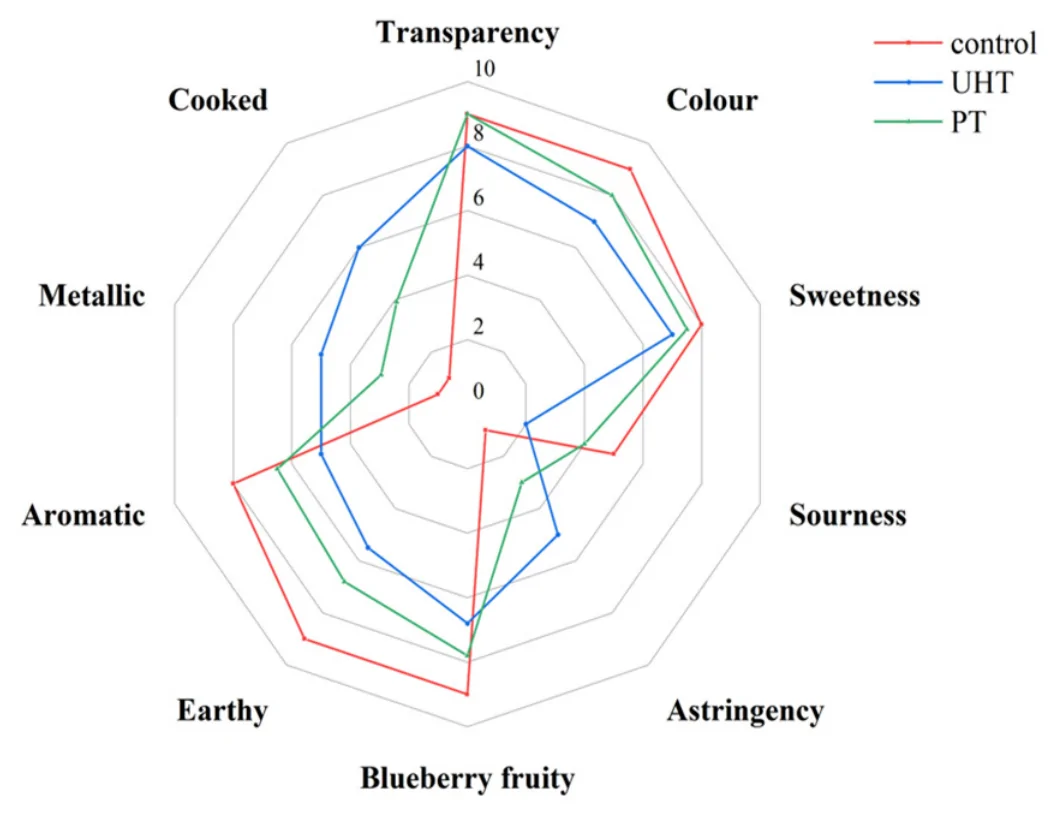
QDA radar plot of blueberry juice treated without thermal processing (Control), treated with PT, and treated with UHT before storage. Ten attributes were scored on a 9-point scale (1 = extremely weak, 9 = extremely strong).

**Figure 7:**
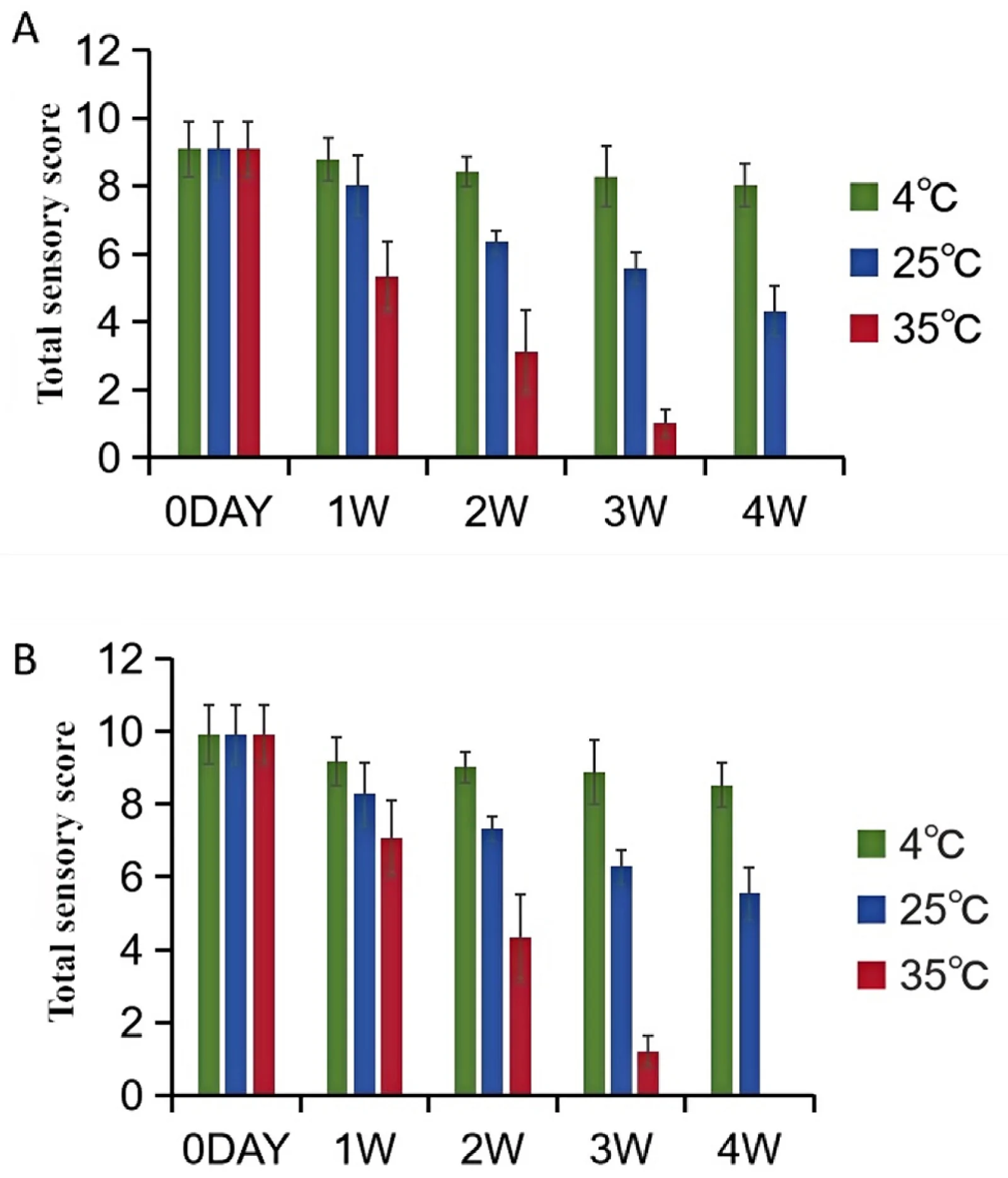
Total sensory evaluation score of blueberry juice stored after UHT (**A**) and PT (**B**) treatments.

Blueberry juice treated with different thermal processing before storage were firstly evaluated by QDA (Figure 6). The results showed that the intensities of blueberry fruity, earthy and floral aromas in blueberry juice decreased after heat treatment, which was consistent with the instrumental quantitative analysis. Substantial losses of terpenoids directly led to a marked reduction in the fruity and floral aromas of blueberry juice, accompanied by an increase in cooked off-flavor. The total sensory scores during storage are shown in Figure 7. Sensory scores gradually decreased with prolonged storage under all temperatures, and higher temperatures accelerated the decrease. The most drastic drop occurred at 35 °C, where the total sensory score reached zero by week 4. At 25 °C, the score decreased to approximately 5 by the end of storage, while at 4 °C, scores remained relatively high after 4 weeks. Compared with UHT-treated juice, PT-treated juice exhibited higher sensory scores at all storage temperatures and time points, including at week 0, indicating that PT caused less initial sensory damage and better maintained sensory quality throughout storage. These sensory results corroborate the instrumental findings [25]. The rapid decline at 35 °C is consistent with the extensive accumulation of off-flavor compounds (furfural and C6 aldehydes) and the near-complete loss of anthocyanins and volatile terpenes observed in preceding sections. The consistently higher scores of PT-treated juice can be attributed to its milder thermal load, which better preserved volatile aroma compounds, bioactive components, and color stability [41]. Low-temperature storage (4 °C) effectively preserved sensory quality, confirming that refrigeration is essential for maintaining the commercial acceptability of thermally processed blueberry juice [34].

## 3. Materials and Methods

### 3.1. Blueberry Juice Preparation and Storage

Fresh blueberries (*Vaccinium* spp., cv. L25, floral-scented) at commercial maturity (12–14 °Brix) were harvested from a plantation in Yunnan Province, China, and transported to the laboratory within 24 h at 4 °C. Damaged fruits were removed. Blueberries were rinsed with sterile distilled water (4 °C), soaked for 5 min with gentle stirring, and blotted dry. The fruits were crushed using a high-speed tissue homogenizer (JJ-2, Changzhou, China) at 12,000 r/min for 30 s. According to Xie et al. [41]. with slight modification, food-grade pectinase (0.07 g/kg) was added to the puree and incubated at 40 °C for 1 h with stirring every 15 min. After cooling to room temperature (25 ± 2 °C), the mixture was vacuum-filtered through 2000-mesh sterile gauze. The filtrate was centrifuged at 2000 r/min and 4 °C for 30 min, and the supernatant was filtered through a 0.22 μm sterile membrane. The clear juice was sealed and stored at 4 °C.

### 3.2. Thermal Processing Treatments

Pasteurization (PT): Sealed samples were preheated to 90 °C and kept in a 90 °C water bath (Memmert GmbH, Schwabach, Germany) for 30 s, then immediately cooled in an ice-water bath to below 25 °C within 2 min. This treatment condition is consistent with the previous literature [42].

Ultra-High Temperature Processing (UHT): A laboratory-scale micro UHT sterilizer (RP-L, Shanghai, China) was used at 135 °C with a holding time of 6 s and a feed flow rate of 5 L/h. The treated juice was aseptically filled into sterile penicillin vials (50 mL) and sealed. The equivalent F_0_ value was calculated as approximately 2.45 min (z = 10 °C, T_ref = 121.1 °C). The UHT condition (135 °C, 6 s) falls within the typical range of 110–138 °C for 2–8 s commonly used for juice processing [4]. Given the acidic nature of blueberry juice (pH 3.24–3.36), this treatment provides sufficient lethality for spoilage microorganisms.

### 3.3. Storage Conditions

Both PT- and UHT-treated juices were stored at 4, 25, and 35 °C in the dark for 28 days. For each storage temperature, sixteen bottles (20 mL per bottle) were prepared, and three bottles were taken weekly for analysis. The temperature was controlled by constant temperature incubators. Samples were collected weekly [43]. A 4-week storage period was selected based on preliminary observations that blueberry juice stored at 35 °C underwent complete sensory deterioration within 4 weeks, consistent with the literature reporting rapid quality loss in processed blueberry products under accelerated storage conditions [43].

### 3.4. Physicochemical Analysis

pH and total soluble solids (TSS) of the juice were measured using a pH meter (FE28, Mettler Toledo, Greifensee, Switzerland) and a refractometer (PAL-1, Atago, Tokyo, Japan).

Color indices (L, a, b*) were measured using a colorimeter (CR-400, Konica Minolta, Tokyo, Japan). Total color difference ΔE* was calculated by the following equation:

$${\Delta E}^{*}=\sqrt{(\Delta L^{*}{)}^{2}+(\Delta a^{*}{)}^{2}+(\Delta b^{*}{)}^{2}}$$

### 3.5. Bioactive Compounds and Antioxidant Activity

To evaluate the effect of thermal processing treatments on bioactive compounds in blueberry juice, total phenolic content (TPC), total flavonoid content (TFC), and total anthocyanin content (TAC) were measured as basic indicators [21,25]. Additionally, DPPH radical scavenging activity was assessed as a preliminary measure of antioxidant capacity [24]. Total phenolic content (TPC) was determined by the Folin–Ciocalteu method [20,25]. Total flavonoid content (TFC) was measured by the aluminum chloride colorimetric method [21,22]. Total anthocyanin content (TAC) was determined by the pH differential method [23]. DPPH radical scavenging activity was measured according to Khatoon et al. [24]. All measurements were performed in triplicate.

### 3.6. Volatile Compound Analysis

Volatile compounds were extracted by headspace solid-phase microextraction (HS-SPME) according to the method of Li et al. [18]. An aliquot of 4 mL juice, 6 mL of saturated NaCl, and 10 μL of 4-octanol (198 mg/L) as internal standard were placed in a 20 mL headspace vial, equilibrated at 50 °C for 10 min, and then extracted with a 2 cm DVB/CAR/PDMS fiber (Supelco, St. Louis, MO, USA) at 50 °C for 20 min. The volatiles were desorbed at 250 °C for 5 min.

A GC 8890-MS 7000D system (Agilent, Santa Clara, CA, USA) equipped with DB-WAX column (30 m × 0.25 mm × 0.25 μm; Agilent, USA) was used. The carrier gas was Helium at 0.9 mL/min. Oven program: 35 °C (5 min) → 1.5 °C/min to 65 °C (2 min) → 3 °C/min to 138 °C (3 min) → 60 °C/min to 230 °C (5 min). EI source at 70 eV, scan range 35–400 amu.

Alkane standards (C10–C40, Sigma-Aldrich, St. Louis, MO, USA) were run on the GC-MS with the same condition described above. Compounds were identified using the NIST20 library (match index > 80) and retention indices. Quantification was performed by internal standard method using 4-octanol.

### 3.7. Analysis of Linalool Enantiomers

Linalool enantiomers were extracted using the same HS-SPME protocol described above. For the enantiomer separation, a Cyclosil-B column (30 m × 0.25 mm × 0.25 μm; Agilent, USA) was used. Other instrumental conditions were the same as Section 2.6.

External standard calibration curves were prepared using (R)-(−)-linalool and (S)-(+)-linalool standards (98% purity, Sigma-Aldrich, USA) at 0.0625–10.00 μg/mL in blank juice matrix with 4-octanol as internal standard [15]. Each measurement was performed in triplicate.

### 3.8. Sensory Evaluation

Sensory evaluations were conducted at the Sensory Laboratory of Huazhong Agricultural University (ethics approval number: HZAUHU-2025-0028). Eight trained panelists (4 male, 4 female) evaluated the samples after 0, 1, 2, 3, and 4 weeks of storage at 4, 25, and 35 °C. The storage conditions of the samples are described in Section 3.3. The blueberry juice samples collected after PT and UHT were firstly evaluated by quantitative descriptive analysis (QDA) with ten attributes (transparency, color, sweetness, sourness, astringency, blueberry fruity, cooked, metallic, earthy, aromatic) on a 9-point scale (1 = extremely weak, 9 = extremely strong). For storage stability evaluation, three composite dimensions—color, aroma, and appearance—were scored according to predefined criteria (Table 4). Color covers visual attributes including transparency. Aroma integrates all olfactory attributes (blueberry fruity, cooked, metallic, earthy, aromatic). Appearance reflects the overall visual integrity (clarity, absence of sedimentation). Weight coefficients (color 0.3434, aroma 0.4485, appearance 0.2081) were determined by a consumer survey (n = 98) [44,45]. The ten sensory attributes and their scoring scales are presented in Table 4, which also shows the initial flavor profiles of the three thermal treatments at week 0.

### 3.9. Statistical Analysis

All experiments were performed in triplicate, and results were expressed as mean ± SD. One-way ANOVA with Tukey’s HSD post hoc test (*p* < 0.05) was conducted using SPSS 27.0. T-tests were used for pairwise comparisons between the PT and UHT treatments at each storage time point, with significance denoted as * (*p* < 0.05), ** (*p* < 0.01), *** (*p* < 0.001), and ns (*p* ≥ 0.05). Figures were generated using GraphPad Prism 9 (GraphPad Software, La Jolla, CA, USA) and Origin 2017 (OriginLab Corporation, Northampton, MA, USA).

## 4. Conclusions

Flavor quality is one of the most important factors affecting consumer acceptance of blueberry juice. To simulate actual production and storage conditions, we investigated the changes in quality parameters of blueberry juice subjected to PT and UHT treatments during storage at 4, 25, and 35 °C for 4 weeks, with special emphasis on bioactive compound retention, volatile changes, and chiral linalool dynamics. The results demonstrated that PT combined with low-temperature storage is a reliable strategy for preserving most quality traits of blueberry juice, as anthocyanins, total phenolics, and antioxidant activity declined slowly under refrigeration. Volatile compounds generally showed a downward trend during storage, but the decline was relatively slow at 4 °C. In contrast, elevated storage temperatures accelerated quality deterioration, characterized by rapid accumulation of furfural, sharp decreases in terpenoids such as linalool, and significant shifts in the R/S enantiomeric ratio of linalool. From a chiral perspective, PT altered the enantiomeric ratio primarily via isomerization of (R)-linalool to (S)-linalool, while UHT caused extensive degradation of (R)-linalool. Low temperature (4 °C) effectively retarded chiral conversion and degradation, preserving the characteristic flavor. The R/S ratio serves as a sensitive indicator of thermal history and storage conditions. Our results showed that the selection of thermal processing treatment and storage temperature jointly determines juice quality, and that PT followed by refrigeration may be a more suitable strategy for the blueberry juice industry. Further research is needed to better understand the mechanism underlying chiral conversion of linalool under different thermal and pH conditions, and to explore approaches for regulating flavor stability of blueberry juice during storage.

## Figures and Tables

**Table 1:** pH and total soluble solids (TSS) of blueberry juice during storage after different thermal processing treatments.

Storage Temperature	Time (Week)	pH (UHT)	pH (PT)	*t*-Test *p* (pH)	TSS (°Brix, UHT)	TSS (°Brix, PT)	*t*-Test *p* (TSS)
	0	3.34 ± 0.02 a	3.34 ± 0.01 a	0.92 (ns)	10.48 ± 0.03 a	10.54 ± 0.06 a	0.41 (ns)
4 °C	1	3.35 ± 0.01 a	3.36 ± 0.02 a	0.85 (ns)	10.43 ± 0.02 a	10.45 ± 0.03 a	0.64 (ns)
	2	3.32 ± 0.02 a	3.33 ± 0.01 a	0.88 (ns)	10.41 ± 0.04 a	10.58 ± 0.03 a	0.13 (ns)
	3	3.34 ± 0.01 a	3.33 ± 0.01 a	0.82 (ns)	10.54 ± 0.03 a	10.53 ± 0.06 a	0.96 (ns)
	4	3.32 ± 0.01 a	3.33 ± 0.01 a	0.91 (ns)	10.55 ± 0.01 a	10.50 ± 0.04 a	0.30 (ns)
25 °C	1	3.35 ± 0.01 a	3.34 ± 0.02 a	0.90 (ns)	10.47 ± 0.05 a	10.45 ± 0.02 a	0.71 (ns)
	2	3.29 ± 0.04 a	3.30 ± 0.02 a	0.91 (ns)	10.30 ± 0.04 a	10.35 ± 0.03 a	0.59 (ns)
	3	3.35 ± 0.01 a	3.32 ± 0.02 a	0.79 (ns)	10.25 ± 0.06 b	10.30 ± 0.06 a	0.80 (ns)
	4	3.33 ± 0.02 a	3.31 ± 0.03 a	0.86 (ns)	10.17 ± 0.01 c	10.22 ± 0.04 b	0.72 (ns)
35 °C	1	3.32 ± 0.06 a	3.32 ± 0.02 a	0.99 (ns)	10.26 ± 0.06 b	10.24 ± 0.04 b	0.79 (ns)
	2	3.31 ± 0.08 a	3.29 ± 0.01 a	0.90 (ns)	10.02 ± 0.02 c	10.01 ± 0.04 c	0.97 (ns)
	3	3.28 ± 0.03 b	3.27 ± 0.04 b	0.86 (ns)	9.82 ± 0.03 d	9.83 ± 0.06 d	0.97 (ns)
	4	3.26 ± 0.07 b	3.24 ± 0.06 b	0.82 (ns)	9.77 ± 0.06 d	9.72 ± 0.03 d	0.67 (ns)

Values are mean ± SD. Different lowercase letters within the same column indicate significant differences (*p* < 0.05, Duncan’s test). The *t*-Test *p* column shows the *p*-value of independent-samples *t*-test comparing UHT versus PT at each time point (ns, not significant (*p* > 0.05)).

**Table 2:** Changes in anthocyanins, total phenolics, total flavonoids and DPPH scavenging activity of blueberry juice during storage after thermal processing treatments.

Storage Temperature	Time (Week)	Anthocyanins (mg CGE/100 mL)			Total Phenolics (mg GAE/100 mL)			DPPH Scavenging Rate (%)			Total Flavonoids (mg RE/100 mL)		
		UHT	PT	*t*-Test *p* (Anthocyanins)	UHT	PT	*t*-Test *p* (Total Phenolics)	UHT	PT	*t*-Test *p* (DPPH Scavenging Rate)	UHT	PT	*t*-Test *p* (Total Flavonoids)
	0	10.12 ± 0.36 a	13.02 ± 0.23 a	0.0003 ***	42.8 ± 0.63 a	55.1 ± 0.29 a	<0.0001 ***	88.37 ± 0.88 a	92.08 ± 1.21 a	0.013 *	38.12 ± 1.26 a	48.83 ± 0.97 a	0.0003 ***
4 °C	1	9.53 ± 1.21 a	11.86 ± 0.12 a	0.029 *	40.43 ± 1.22 ab	51.45 ± 0.13 a	0.0001 ***	87.32 ± 1.99 a	89.12 ± 1.45 a	0.274 ns	37.32 ± 2.16 a	46.32 ± 1.25 a	0.003 **
	2	8.95 ± 0.32 b	10.33 ± 0.11 a	0.002 **	38.41 ± 1.34 b	49.58 ± 0.33 b	0.0002 ***	87.02 ± 2.16 a	85.02 ± 1.66 b	0.272 ns	36.08 ± 1.26 b	43.82 ± 2.01 b	0.005 **
	3	8.34 ± 0.11 bc	9.88 ± 0.11 ab	<0.0001 ***	38.54 ± 1.13 b	47.17 ± 1.26 bc	0.0009 ***	88.16 ± 3.86 a	81.23 ± 1.85 c	0.049 *	35.12 ± 0.82 b	42.02 ± 1.53 b	0.002 **
	4	6.92 ± 0.15 c	9.42 ± 0.25 b	0.0001 ***	33.38 ± 0.69 c	42.97 ± 1.28 c	0.003 **	84.43 ± 2.21 b	82.62 ± 2.22 c	0.374 (ns)	33.18 ± 2.07 c	39.21 ± 1.36 c	0.014 *
25 °C	1	9.02 ± 0.01 b	11.93 ± 0.32 a	0.0001 ***	38.47 ± 0.25 b	50.45 ± 2.02 b	0.0005 ***	86.98 ± 2.13 a	88.37 ± 1.57 a	0.414 (ns)	36.82 ± 1.14 a	44.76 ± 1.03 b	0.0009 ***
	2	8.29 ± 2.04 bc	10.30 ± 0.52 a	0.174 (ns)	34.30 ± 1.34 c	46.38 ± 3.03 bc	0.003 **	83.13 ± 1.66 b	85.32 ± 2.01 b	0.219 (ns)	32.16 ± 0.29 b	40.18 ± 0.91 c	0.0001 ***
	3	6.35 ± 1.01 d	8.35 ± 0.32 b	0.031 *	30.25 ± 2.46 d	41.22 ± 2.46 d	0.005 **	80.01 ± 2.12 bc	82.32 ± 1.66 c	0.211 (ns)	28.88 ± 2.21 c	36.32 ± 2.21 d	0.015 *
	4	6.16 ± 1.06 d	7.81 ± 0.63 c	0.081 (ns)	29.33 ± 2.42 d	39.28 ± 1.68 d	0.004 **	82.06 ± 1.22 b	84.02 ± 2.03 c	0.225 (ns)	27.36 ± 1.61 c	34.27 ± 0.88 d	0.003 **
35 °C	1	9.26 ± 0.13 ab	11.26 ± 0.03 a	<0.0001 ***	37.08 ± 0.38 c	42.33 ± 0.57 d	0.0002 ***	82.23 ± 0.81 bc	80.68 ± 0.51 c	0.049 *	35.27 ± 0.92 b	41.02 ± 0.72 c	0.001 **
	2	8.02 ± 1.02 bc	10.11 ± 0.01 a	0.024 *	36.06 ± 0.58 c	38.82 ± 1.23 e	0.025 *	82.07 ± 0.67 bc	80.09 ± 0.66 c	0.022 *	31.43 ± 1.55 c	37.32 ± 1.22 d	0.007 **
	3	6.24 ± 0.16 d	8.86 ± 0.02 b	<0.0001 ***	35.43 ± 1.62 c	38.66 ± 2.51 e	0.134 (ns)	75.44 ± 2.72 c	72.66 ± 3.61 d	0.347 (ns)	28.32 ± 2.01 d	32.07 ± 0.82 e	0.040 *
	4	6.08 ± 0.24 d	7.47 ± 0.78 c	0.042 *	32.26 ± 1.61 d	33.01 ± 3.61 f	0.759 (ns)	74.30 ± 4.74 c	73.78 ± 5.31 d	0.905 (ns)	25.27 ± 1.34 d	29.92 ± 1.72 f	0.021 *

Values are expressed as mean ± standard deviation (*n* = 3). Different lowercase letters (a–f) within the same column indicate significant differences among storage time points/temperatures within the same treatment, as determined by one-way ANOVA followed by Duncan’s multiple range test (*p* < 0.05). The *p*-values shown in the columns headed “*t*-test *p*” were obtained by independent-samples Student’s *t*-test comparing UHT and PT treatments at each corresponding storage time point; *, ** and *** denote significant differences at *p* < 0.05, *p* < 0.01 and *p* < 0.001, respectively, and ns indicates no significant difference (*p* ≥ 0.05).

**Table 3:** Enantiomeric ratio of (R)-linalool to (S)-linalool in blueberry juice during storage.

Storage Temperature	Time (Week)	Control	UHT	PT
	0	94:6 a	75:25 c	90:10 b
4 °C	1	92:8 a	71:29 c	86:14 b
	2	90:10 a	69:31 c	83:17 b
	3	89:11 a	68:32 c	82:18 b
	4	89:11 a	67:33 c	81:19 b
25 °C	1	91:9 a	57:43 c	87:13 b
	2	87:13 a	54:46 c	83:17 b
	3	85:15 a	46:54 c	80:20 b
	4	82:18 a	36:64 c	75:25 b
35 °C	1	88:12 a	50:50 c	83:17 b
	2	84:16 a	41:59 c	78:22 b
	3	78:22 a	33:67 c	68:32 b
	4	70:30 a	27:73 c	62:38 b

Enantiomeric ratios of (R)-linalool to (S)-linalool (R:S) are presented. Different lowercase letters within the same column indicate significant differences (*p* < 0.05, Duncan’s test).

**Table 4:** Sensory evaluation criteria for blueberry juice.

Attribute	Grade	Description	Score
Color	Excellent	Uniform and bright	8–10
	Good	Uniform but slightly dull	5–7
	Fair	Dull and slightly turbid	2–4
	Poor	Dark and turbid	0–1
Aroma	Excellent	Characteristic blueberry aroma, rich	8–10
	Good	Characteristic aroma present but weak	5–7
	Fair	Weak aroma	2–4
	Poor	Off-odor	0–1
Appearance	Excellent	Homogeneous, no sediment	8–10
	Good	Slight flocculation	5–7
	Fair	Some sedimentation	2–4
	Poor	Heavy sedimentation	0–1

## Data Availability

The data presented in this study are available on request from the corresponding author. The data are not publicly available due to privacy or ethical restrictions.

## Supplementary Materials

The following supporting information can be downloaded at https://www.mdpi.com/article/10.3390/molecules31183198/s1. Table S1. Concentrations and odor activity values (OAVs) of volatile compounds in blueberry juice at week 0; Table S2. Changes in volatile compound concentrations (μg/L) in UHT-treated blueberry juice during storage at 4, 25, and 35 °C. Table S3. Changes in volatile compound concentrations (μg/L) in PT-treated blueberry juice during storage at 4, 25, and 35 °C.
