# Supplementary material for: Impact of Thermal Processing and Storage on the Quality and Chirality of Linalool in Blueberry Juice
Source: Molecules. 2026 Sep 10;31(18):3198. doi: 10.3390/molecules31183198 (PMC13609378; doi:10.3390/molecules31183198)
Supplement: Supplementary file 1 [file molecules-31-03198-s001.zip › molecules-4494161-supplementary.pdf]

Supplementary Materials

Table S1. Concentrations and odor activity values (OAVs) of volatile compounds in blueberry juice at week 0.

Volatile compound	Threshold (μg/L)	Control (μg/L)	UHT (μg/L)	PT (μg/L)	Control OAV	UHT OAV	PT OAV
**Esters (total)**		9.76	1.34	5.03			
Ethyl acetate	5	3.57±0.06	0.84±0.18	2.29±0.34	0.71	0.17	0.46
2-Methylbutyrate ethyl ester	0.1	2.00±0.12	0.27±0.10	0.97±0.05	20.00	2.67	9.73
Methyl isovalerate	4.4	2.31±0.12	0.23±0.09	1.22±0.12	0.53	0.05	0.28
Methyl 2-methylbutyrate	0.4	1.06±0.17	ND	ND	2.66	0	0
Ethyl hexanoate	2.2	0.82±0.17	0	0.54±0.11	0.37	0	0.25
**Terpenes (total)**		742.00	338.04	552.19			
Myrcene	12	31.80±3.47	10.66±1.60	22.57±1.52	0.25	0.89	2.65
α-Terpinene	15	1.94±0.17	ND	0.19±0.04	0.13	0	0.01
D-Limonene	20	10.85±1.48	2.40±0.54	6.41±0.94	0.54	0.12	0.32
Eucalyptol	1.3	9.58±0.75	2.10±0.13	5.66±0.68	7.37	1.62	4.36
Terpinolene	200	2.12±0.14	ND	0.74±0.06	0.01	0	0
(Z)-Linalool oxide	100	47.80±2.14	94.90±6.80	65.78±6.11	0.48	0.95	0.66
(E)-Linalool oxide	190	27.37±3.48	73.61±5.22	50.93±2.25	0.14	0.39	0.27
Linalool	6	300.14±9.25	72.43±5.17	206.90±6.73	50.00	12.07	34.48
Geraniol	130	116.38±6.61	35.26±5.86	65.91±6.12	0.90	0.27	0.51
4-Terpineol	5	4.44±0.40	ND	2.09±0.15	0.89	0	0.42
Neral	40	1.56±0.32	ND	0.56±0.27	0.04	0	0.01
α-Terpineol	330	156.92±13.96	39.85±3.88	107.50±8.38	0.48	0.12	0.33
β-Citronellol	62	0.89±0.15	ND	0	0.01	0	0
Geranial	60	6.31±0.42	0.82±0.18	2.98±0.21	0.11	0.01	0.05
Nerol	50	20.96±4.11	6.01±0.67	13.43±1.91	0.42	0.12	0.27
β-Damascenone	1.3	0.64±0.10	ND	0	0.50	0	0
Myrtenol	7	0.61±0.16	ND	0	0.09	0	0
Carveol	250	1.68±0.33	ND	0.56±0.20	0.01	0	0
**Ketones (total)**		12.37	38.00	22.84			
Geranylacetone	60	5.04±0.18	13.92±0.66	7.78±0.45	0.08	0.23	0.13
2-Heptanone	6.8	1.92±0.16	8.70±0.45	4.63±0.40	0.28	1.28	0.68
6-Methyl-5-hepten-2-one	50	5.41±1.16	15.37±0.95	10.43±0.73	0.11	0.31	0.21
Alcohols (total)		491.32	141.86	301.22			
1-Hexanol	6	219.04±17.65	21.51±1.35	99.24±5.83	36.51	3.59	16.54
Benzyl alcohol	2546	67.10±5.75	30.00±3.07	46.59±6.13	0.03	0.01	0.02
Phenylethyl alcohol	1000	105.92±2.81	74.05±6.51	98.74±3.50	0.11	0.07	0.10
(E)-2-Hexenol	50	94.95±9.39	16.30±3.54	54.62±7.63	1.90	0.33	1.09
(E)-3-Hexen-1-ol	1000	4.31±1.03	ND	2.03±0.85	0	0	0
**Phenols (total)**		4.59	4.97	4.84			
Eugenol	26	4.59±0.51	4.97±0.13	4.84±0.28	0.18	0.19	0.19
**Aldehydes (total)**		220.75	216.08	132.87			
Hexanal	4.5	84.46±6.11	16.62±6.09	43.59±6.00	18.77	3.69	9.69
(E)-2-Hexenal	17	125.08±11.46	16.62±5.35	76.17±6.01	7.36	0.98	4.48
Benzaldehyde	750	1.16±0.24	ND	0.94±0.26	0	0	0
Phenylacetaldehyde	105	4.11±0.20	0.74±0.06	1.90±0.19	0.04	0.01	0.02
Heptanal	3	2.95±0.43	0.17±0.11	1.48±0.49	0.98	0.06	0.49
Nonanal	0.34	2.55±0.52	ND	2.51±0.49	7.51	0	7.38
Furfural	3500	0	131.30±3.87	3.75±0.80	0	0.04	0
Isovaleraldehyde	16.5	0	37.42±5.77	1.15±0.07	0	2.27	0.07
(E)-2-Octenal	4	0.44±0.13	13.26±0.91	1.38±0.12	0.11	3.31	0.35

Note: Data are expressed as mean ± standard deviation. One-way analysis of variance (ANOVA) with Duncan's multiple comparison test was used to determine significant differences ($p < 0.05$). Bold values represent total amounts of each compound class. Compound concentrations and odor thresholds are expressed in μg/L; odor thresholds were referenced from Cheng Y et al. (2022). ND: not detected.

Table S2. Changes in volatile compound concentrations (μg/L) in UHT-treated blueberry juice during storage at 4, 25, and 35 °C.

Compound	Week 0	4°C W1	4°C W2	4°C W3	4°C W4	25°C W1	25°C W2	25°C W3	25°C W4	35°C W1	35°C W2	35°C W3	35°C W4
Ethyl acetate	0.84	0.81	0.82	0.83	0.86	0.62	0.41	0.32	0.21	0.51	0.43	0.21	0.11
2-Methylbutyrate ethyl ester	0.27	0.26	0.28	0.31	0.26	0.25	0.15	0.12	0.06	0.12	0.03	0	0
Methyl isovalerate	0.23	0.22	0.22	0.21	0.2	0.21	0.15	0.12	0.03	0.2	0.08	0.02	0
Methyl 2-methylbutyrate	0	0	0	0	0	0	0	0	0	0	0	0	0
Ethyl hexanoate	0	0	0	0	0	0	0	0	0	0	0	0	0
Myrcene	10.66	10.53	10.28	10.06	9.83	9.33	8.76	8.21	7.98	8.21	4.33	1.67	0
α-Terpinene	0	0	0	0	0	0	0	0	0	0	0	0	0
D-Limonene	2.4	2.32	2.21	2.15	2.03	2.1	1.91	1.69	1.53	1.76	1.02	0.68	0
Eucalyptol	2.1	2.09	1.99	2.08	1.98	1.84	1.61	1.25	1.12	1.58	1.11	0.83	0.21
Terpinolene	0	0	0	0	0	0	0	0	0	0	0	0	0
cis-Linalool oxide	94.9	90.16	86.21	79.68	76.32	83.04	69.28	49.31	32.45	71.18	37.45	23.73	6.75
trans-Linalool oxide	73.61	69.93	66.25	64.57	60.89	64.41	57.32	49.01	41.32	55.21	41.8	24.4	11.2
Linalool	72.43	68.81	65.19	61.57	57.94	65.38	59.32	51.27	46.22	54.32	28.22	11.11	2.26
Geraniol	35.26	34.5	32.73	30.97	29.88	30.85	27.45	21.04	14.32	26.45	11.63	3.81	0
4-Terpineol	0	0	0	0	0	0	0	0	0	0	0	0	0
Neral	0	0	0	0	0	0	0	0	0	0	0	0	0
α-Terpineol	39.85	37.86	35.87	33.87	31.88	34.27	30.89	26.91	21.93	29.89	19.95	10.18	2.38
β-Citronellol	0	0	0	0	0	0	0	0	0	0	0	0	0
Geranial	0.82	0.78	0.74	0.7	0.66	0.72	0.61	0.51	0.41	0.61	0.41	0.2	0
Nerol	6.01	5.82	5.33	5.09	4.91	5.26	4.81	4.16	3.64	4.51	3.38	1.72	0.28
β-Damascenone	0	0	0	0	0	0	0	0	0	0	0	0	0
Myrtenol	0	0	0	0	0	0	0	0	0	0	0	0	0
Carveol	0	0	0	0	0	0	0	0	0	0	0	0	0
Geranylacetone	13.92	14.62	15.07	15.72	16.02	15.66	18.33	22.83	25.57	19.4	23.88	30.36	33.84
2-Heptanone	8.7	9.13	9.57	10.0	10.44	9.79	11.88	13.96	16.05	13.88	15.05	18.22	21.4
6-Methyl-5-hepten-2-one	15.37	16.14	16.91	17.28	17.99	17.29	21.21	23.13	25.05	20.21	25.05	32.9	36.74
1-Hexanol	21.51	21.29	21.08	20.86	20.65	20.32	18.45	17.56	14.44	18.18	15.32	11.11	9.82
Benzyl alcohol	30.0	29.7	29.25	28.97	28.25	28.35	26.5	23.75	21.07	27.35	23.11	20.39	18.73
Phenylethyl alcohol	74.05	73.31	72.57	71.83	71.09	71.2	68.35	63.5	59.64	70.35	66.64	59.94	53.24
(E)-2-Hexenol	16.3	16.14	15.97	15.92	15.65	15.89	14.93	13.27	11.67	13.28	11.67	8.86	6.04
(E)-3-Hexen-1-ol	0	0	0	0	0	0	0	0	0	0	0	0	0
Eugenol	4.97	4.99	4.95	5.01	4.92	5.03	5.09	5.16	5.22	5.11	5.32	5.41	5.72
Hexanal	16.62	17.25	18.08	19.61	20.86	19.34	23.87	28.59	31.92	23.27	32.92	38.56	46.21
(E)-2-Hexenal	16.62	17.95	19.28	20.61	21.94	19.94	22.77	27.59	31.92	23.27	29.92	36.56	43.21
Benzaldehyde	0	0	0	0	0	0	0	0	0	0	0	0	0
Phenylacetaldehyde	0.74	0.8	0.82	0.91	0.95	0.89	1.15	1.23	1.46	1.04	1.33	1.83	2.12
Heptanal	0.17	0.21	0.16	0.15	0.17	0.26	0.36	0.78	1.02	0.45	0.76	1.08	1.44
Nonanal	0	0	0	0	0	0	0	0	0	0	0	0	0
Furfural	131.25	128.75	132.25	142.75	145.25	157.5	163.75	190.25	216.25	183.75	206.25	238.75	260.25
Isovaleraldehyde	37.42	39.41	42.41	44.4	45.39	44.92	50.39	57.87	65.36	56.39	72.36	81.32	93.29
(E)-2-Octenal	13.26	14.32	15.02	15.83	16.02	15.91	20.56	23.22	26.87	22.56	27.87	32.17	43.48

Note: W1–W4 denote storage weeks 1–4. Data are expressed in μg/L. ND: not detected.

Table S3. Changes in volatile compound concentrations (μg/L) in PT-treated blueberry juice during storage at 4, 25, and 35 °C.

Compound	Week 0	4°C W1	4°C W2	4°C W3	4°C W4	25°C W1	25°C W2	25°C W3	25°C W4	35°C W1	35°C W2	35°C W3	35°C W4
Ethyl acetate	2.29	2.2	2.13	2.07	2.02	2.02	1.95	1.87	1.6	1.95	1.605	1.26	0.92
2-Methylbutyrate ethyl ester	0.97	0.94	0.91	0.88	0.85	0.9	0.82	0.75	0.68	0.82	0.675	0.53	0.39
Methyl isovalerate	1.22	1.18	1.15	1.11	1.07	1.13	1.04	0.95	0.85	1.04	0.855	0.67	0.49
Methyl 2-methylbutyrate	0	0	0	0	0	0	0	0	0	0	0	0	0
Ethyl hexanoate	0.54	0.52	0.51	0.49	0.48	0.5	0.46	0.42	0.38	0.46	0.38	0.3	0.22
Myrcene	22.57	21.44	20.31	19.18	18.06	19.75	15.93	14.11	11.29	16.93	14.465	12.0	9.5
α-Terpinene	0.19	0.21	0.2	0.15	0.22	0.14	0.15	0.21	0.2	0.18	0.2	0.22	0.23
D-Limonene	6.41	6.09	5.77	5.65	5.53	5.61	4.81	4.01	3.21	4.81	3.205	1.6	0
Eucalyptol	5.66	5.38	5.39	5.02	5.12	4.95	4.75	4.54	3.83	3.25	2.335	1.42	0
Terpinolene	0.74	0.77	0.63	0.75	0.74	0.63	0.76	0.75	0.76	0.69	0.715	0.74	0.73
cis-Linalool oxide	65.78	62.49	69.2	65.91	62.62	57.56	50.34	43.11	36.89	39.34	22.895	6.45	0
trans-Linalool oxide	50.93	48.38	47.84	45.29	43.74	46.56	39.2	33.83	28.46	38.2	25.465	12.73	0
Linalool	206.89	196.55	198.2	185.86	176.51	179.03	162.17	149.31	121.44	155.17	140.085	125.0	110.0
Geraniol	65.91	62.61	59.32	58.02	57.73	58.67	54.43	49.19	42.95	49.43	32.955	16.48	8.16
4-Terpineol	2.09	2.32	2.15	2.03	2.11	1.98	2.03	2.21	2.05	2.08	2.1	2.12	2.04
Neral	0.56	0.66	0.43	0.52	0.59	0.51	0.47	0.62	0.56	0	0	0	0
α-Terpineol	107.49	102.12	98.74	95.37	93.99	94.05	85.62	78.18	73.74	80.62	72.31	64.0	56.0
β-Citronellol	0	0	0	0	0	0	0	0	0	0	0	0	0
Geranial	2.98	2.83	2.68	2.53	2.38	2.91	2.33	1.98	1.77	2.23	1.485	0.74	0
Nerol	13.43	12.76	12.09	11.42	11.74	12.25	11.07	10.39	9.91	10.07	8.215	6.36	3.28
β-Damascenone	0	0	0	0	0	0	0	0	0	0	0	0	0
Myrtenol	0	0	0	0	0	0	0	0	0	0	0	0	0
Carveol	0.56	0.56	0.46	0.66	0.52	0.54	0.49	0.52	0.53	0.04	0.02	0	0
Geranylacetone	7.78	8.17	8.56	8.95	9.34	8.75	10.72	15.7	20.67	13.72	17.86	22.0	26.0
2-Heptanone	4.63	4.86	5.09	5.32	5.56	6.21	7.79	9.37	12.95	7.79	10.145	12.5	15.0
6-Methyl-5-hepten-2-one	10.43	10.95	11.08	11.22	11.87	13.73	15.04	18.34	25.64	18.04	23.02	28.0	33.0
1-Hexanol	99.24	106.68	114.13	121.57	129.01	117.85	126.45	145.06	169.67	136.45	150.725	165.0	180.0
Benzyl alcohol	46.59	50.08	53.58	57.07	60.57	55.33	64.06	72.8	81.53	64.06	81.53	99.0	116.48
Phenylethyl alcohol	98.74	106.15	113.55	120.96	128.36	117.25	135.77	154.28	172.79	135.77	150.385	165.0	180.0
(E)-2-Hexenol	54.62	58.72	62.81	66.91	71.01	68.86	86.1	98.34	105.58	85.1	95.05	105.0	115.0
(E)-3-Hexen-1-ol	2.03	2.18	2.33	2.49	2.64	2.41	2.79	3.17	3.55	2.79	3.55	4.31	5.07
Eugenol	4.84	4.86	4.89	4.91	4.88	4.91	4.86	5.02	4.92	4.96	5.08	5.2	5.32
Hexanal	43.59	47.08	50.56	54.05	57.54	55.31	67.03	75.74	79.46	61.03	83.465	105.9	133.33
(E)-2-Hexenal	76.17	82.26	90.36	95.45	102.54	95.4	110.64	121.87	137.11	106.64	137.105	167.57	198.04
Benzaldehyde	0.94	0.96	1.02	0.91	0.95	0.98	1.04	0.93	0.89	0.94	0.985	1.03	0.93
Phenylacetaldehyde	1.9	2.05	2.2	2.36	2.51	2.32	2.86	3.14	3.42	2.86	3.52	4.18	5.33
Heptanal	1.48	1.6	1.81	1.89	1.95	2.32	2.57	3.02	3.22	2.67	2.995	3.32	4.88
Nonanal	2.51	2.49	2.66	2.51	2.52	2.55	3.48	3.98	4.26	3.22	4.11	5.0	6.0
Furfural	3.75	3.81	3.87	3.93	3.99	3.98	4.35	4.92	5.35	5.05	6.275	7.5	8.8
Isovaleraldehyde	1.15	1.17	1.19	1.21	1.22	1.32	1.48	1.59	1.66	1.44	1.685	1.93	2.12
(E)-2-Octenal	1.38	1.49	1.6	1.71	1.82	1.66	1.93	2.45	2.98	1.96	2.43	2.9	3.4

Note: W1–W4 denote storage weeks 1–4. Data are expressed in μg/L. ND: not detected.
